# HHV-6B ribonucleotide reductase sequesters NF-κB subunit p65 to inhibit innate immune responses

**DOI:** 10.1016/j.isci.2024.111710

**Published:** 2024-12-30

**Authors:** Mansaku Hirai, Khoir Amaliin, Jing Rin Huang, Salma Aktar, Yasuko Mori, Jun Arii

**Affiliations:** 1 Division of Clinical Virology, Center for Infectious Diseases, Kobe University Graduate School of Medicine, Kobe, Hyogo, Japan

**Keywords:** Cell biology, Immunology, Virology

## Abstract

Human herpesvirus 6B (HHV-6B) belongs to the genus *Roseolovirus* of the betaherpesvirus subfamily, causing exanthema subitum and encephalitis. Although viral ribonucleotide reductase (RNR) is conserved in betaherpesviruses, it has lost its enzymatic activity. Human cytomegalovirus (HCMV) belongs to the other betaherpesvirus genus, *Cytomegalovirus*; its RNR inhibits nuclear factor-kappa B (NF-κB) signaling via interaction with the adaptor molecule RIPK1. However, the significance of enzymatically inactive RNR in roseoviruses is unclear. Here, we show that the RNRs from all three human roseoloviruses inhibit NF-κB activation. HHV-6B RNR sequesters NF-κB subunit p65 in the cytoplasm and inhibits its translocation into the nucleus. Silencing HHV-6B RNR increased the expression of inflammatory molecules in infected cells. This study reveals that inhibiting NF-κB is a conserved role of the RNR in betaherpesviruses but that the precise mechanisms responsible for these effects are different.

## Highlights

• HHV-6B ribonucleotide reductase (RNR) inhibits NF-κB activation
• HHV-6B RNR sequesters NF-κB p65 to inhibit its translocation into the nucleus
• HHV-6B RNR represses the expression of inflammatory molecules in infected cells
• Inhibiting NF-κB is a conserved but its mechanisms are different in betaherpesviruses

## Graphical abstract

**Figure:**
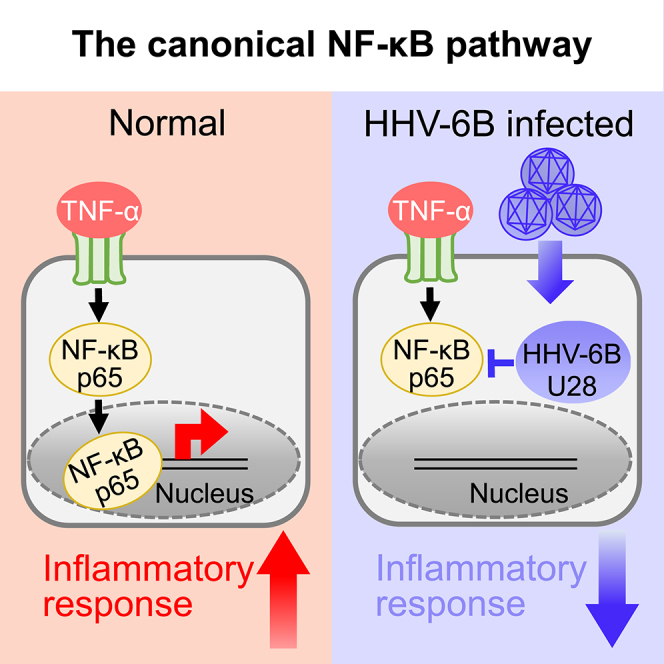

## Introduction

Herpesviruses are enveloped double-stranded DNA viruses which cause life-long latent infection in the host. The *Herpesviridae* family is subdivided into the *Alphaherpesvirinae*, *Betaherpesvirinae*, and *Gammaherpesvirinae* subfamilies, based on their molecular and biological properties.^1^ Briefly, they establish latency in neurons, hematopoietic progenitor cells, or B cells, respectively.^1^ Of these, betaherpesviruses including human cytomegalovirus (HCMV) and human herpesvirus 6B (HHV-6B) are a major cause of opportunistic infections that can lead to life-threatening complications in immunocompromized individuals.^2^^,^^3^ HHV-6B and related viruses, such as HHV-6A and HHV-7 belong to the *Roseolovirus* genus within the *Betaherpesvirinae* subfamily.^3^ Their replication is restricted to T lymphocytes.^3^ In contrast, HCMV can replicate in many cell types but not in lymphocytes.^2^ HHV-6B infects nearly 90% of the world’s population in the first 2 years of life and is responsible for exanthema subitum, a pathology defined by skin rashes, high fever, and respiratory distress.^3^ Like other herpesviruses, HHV-6B establishes lifelong latent infection in humans and can reactivate to cause encephalitis especially in individuals with compromised immunity.^3^

**Figure 1:**
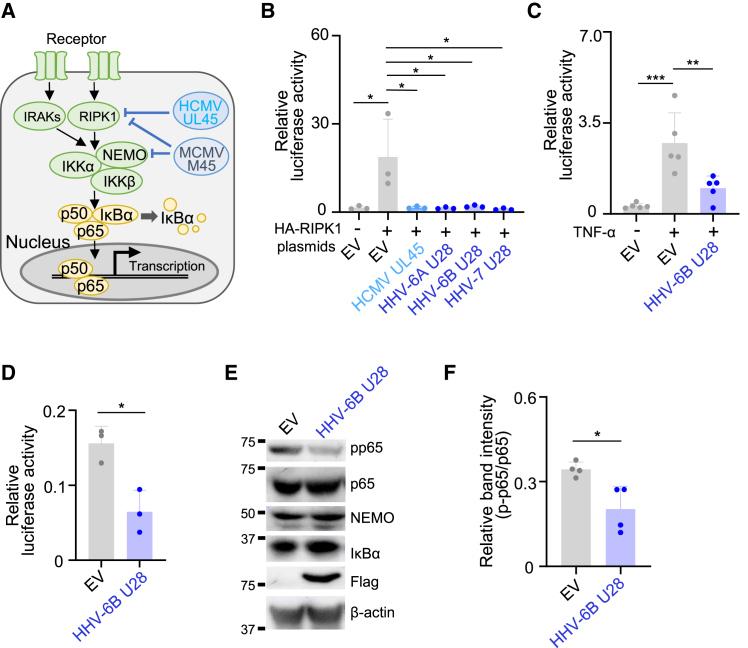
Roseolovirus U28 proteins inhibit NF-κB activation (A) Schematic representation of NF-κB-activating signaling pathways. HCMV UL45 targets RIPK1 whereas MCMV M45 degrades RIPK1 and NEMO. (B and C) HEK293T cells were co-transfected with a plasmid encoding firefly luciferase under the control of the NF-κB promoter (NF-κB luc), pRL-SV40 as an internal control reporter and the indicated expression plasmids or empty vector (EV), together with or without HA-RIPK1. The luciferase activity was measured 24 h post-transfection. The data are shown as means and standard deviations (*n* = 3; ∗, *p* < 0.05 [Tukey’s test]) (C) HEK293T cells were transfected with the same plasmid described in (B) without HA-RIPK1. After 24 h, the cells were incubated with 20 ng/mL of TNF-α for 4 h, followed by luciferase assay. The data are shown as means and standard deviations (*n* = 5; ∗∗, *p* < 0.01; ∗∗∗, *p* < 0.001 [Tukey’s test]). (D) HEK293T cells were transfected with NF-κB-luc plasmid and pRL-SV40 together with FLAG-HHV-6B U28 expression plasmid or empty vector (EV). The luciferase activity was measured 24 h post-transfection. The data are shown as means and standard deviations (*n* = 3; ∗, *p* < 0.05 [Welch’s t test]). (E) HEK293T cells were transfected with the FLAG-HHV-6B U28 expression plasmid or empty vector (EV). After 48 h, the cells were analyzed by immunoblotting with the indicated antibodies. Phosphorylated p65 at Ser-536 was indicated as pp65. (F) The intensities of each protein in (E) normalized to p65 are shown as means with standard deviations (*n* = 4; ∗, *p* < 0.05 [Welch’s t test]). See also Figure S1.

The nuclear factor-kappa B (NF-κB) transcription factor is a critical regulator of the host cell’s early response to viral infection^4^ which induces the transcriptional activity of NF-κB. This subsequently drives expression of a number of different proinflammatory cytokines as well as viral genes.^4^^,^^5^ NF-κB consists of a heterodimer of p65 and p50 present in the cytoplasm bound to the inhibitor of NF-κB proteins, IκBα (Figure 1A). The canonical NF-κB pathway is regulated by a cascade of events that initiates upon receptor stimulation and recruitment of receptor-interacting protein kinase 1 (RIPK1) or the adaptor proteins interleukin receptor-associated kinases (IRAKs), followed by activation of the IκB kinase (IKK) complex. The IKK complex consists of two catalytic subunits, IKKα and β, and NF-κB essential modulator (NEMO). The activated IKK complex phosphorylates IκBα, which is then polyubiquitinated, resulting in its proteasomal degradation.^4^ Subsequently, the NF-κB p65/p50 heterodimer is released from cytoplasmic sequestration and translocates into the nucleus, where it binds to the promoters of downstream genes and initiates their expression.^4^ During this process, NF-κB p65 serine at position 536 (Ser-536) in the transactivation domain is phosphorylated by various protein kinases including IKKs, which results in enhancement of its transactivation activity.^4^^,^^5^ There is increasing evidence that viruses can inhibit NF-κB signaling by interacting with components of the NF-κB cascade.^6^^,^^7^

Viral ribonucleotide reductase (RNR) large subunit (R1) is conserved in *Herpesviridae* (Figure S1A). It has been well-established that RNR, which consists of R1 and the small subunit (R2), supplies deoxyribonucleotides and enhances viral genome replication in some herpesviruses.^8^ In addition, herpesvirus RNR R1s are known to have a variety of functions other than in nucleic acid metabolism. The RNR R1s of herpes simplex virus 1 (HSV-1) (UL39) and murine cytomegalovirus (MCMV) (M45) act as suppressors of necroptosis by inhibiting interactions between RIPK1 and RIPK3, a key step in this process.^9^^,^^10^^,^^11^ RNR R1s of alpha- or gammaherpesviruses are known to inhibit the antiviral factor APOBEC3.^12^^,^^13^ RNR R1 is also conserved in betaherpesviruses but has lost its enzymatic activity.^8^ RNR R2 is also encoded in alpha- and gammaherpesvirus genomes, whereas betaherpevirus genomes lack RNR R2. Thus, genome replication of betaherpesviruses depends on deoxyribonucleotides supplied by cellular RNR.^8^ Furthermore, the RNR R1 of HCMV (UL45) does not impair programmed cell death and restriction by APOBEC3^14^^,^^15^ but is required for optimal viral replication and suppression of RIPK1-mediated NF-κB signaling at the late stages of viral infection in cooperation with viral deubiquitinase UL48.^16^^,^^17^ Impairment of NF-κB signaling by viral RNR R1 might be common in betaherpesviruses as MCMV M45 inhibits RIPK1-mediated signaling pathways, including activation of NF-κB after stimulation via the tumor necrosis factor receptor 1 (TNFR1) or Toll-like receptor 3 (TLR3).^18^ MCMV M45 also induces lysosomal degradation of NEMO to block RIPK1-independent NF-κB activation^19^^,^^20^ (Figure 1A). However, the significance of the enzymatically inactive RNR in roseoviruses is unclear. In this study, we analyzed the function of the HHV-6B (U28) RNR R1 and found that it inhibits NF-κB activity via interaction with p65.

## Results

### Roseolovirus U28 inhibits NF-κB activation

To determine whether HHV-6B RNR R1 (U28) affects NF-κB signaling, HEK293T cells were co-transfected with plasmids expressing individual RNR R1s of human betaherpesviruses together with HA-tagged RIPK1 and NF-κB response element firefly luciferase reporter plasmid (NF-κB-luc) (Figures 1B and S1B). As expected, ectopic expression of RIPK1 enhanced NF-κB activity. Expression of HCMV RNR R1 (UL45) effectively inhibited RIPK1-mediated NF-κB activation (Figure 1B), consistent with a previous report.^17^ Furthermore, expression of RNR R1 (U28) of HHV-6A, HHV-6B or HHV-7 also significantly inhibited NF-κB activation. Additionally, NF-κB activation induced by tumor necrosis factor α (TNF-α) treatment through TNFR1 and RIPK1 was inhibited by expression of U28 of HHV-6B (Figure 1C). These results indicate that inhibition of NF-κB activation is a conserved function of human betaherpesviruses.

During canonical NF-κB signaling, after degradation of IκBα a dimeric complex comprising the NF-κB subunits p65 and p50 translocates to the nucleus and activates gene transcription.^4^ Degradation of IκBα is initiated by its phosphorylation by the IKK complex consisting of IKKα, IKKβ, and NEMO. Although the NF-κB cascade is activated in response to infection, it is also important for cell survival and proliferation. Without any stimulation, HEK293T cells maintain NF-κB signaling at a low level, and therefore, we analyzed cells expressing HHV-6B U28 without any stimulation. HEK293T cells were co-transfected with a plasmid expressing FLAG-HHV-6B U28 or control plasmid coupled with NF-κB-luc, and luciferase assays were performed. Luciferase activity was detected without any stimulation but at a lower level than in HA-RIPK1-transfected cells (Figures 1B and 1D). Expression of HHV-6B U28 significantly reduced luciferase activity relative to the control (Figure 1D), suggesting that it inhibits the endogenous activity of NF-κB in HEK293T cells. Next, HEK293T cells transfected with the FLAG-HHV-6B U28 plasmid were analyzed by immunoblotting 48 h thereafter. Expression of HHV-6B U28 did not change the amount of p65, NEMO, or IκBα (Figures 1E and S1C). In contrast, phosphorylation of p65 Ser-536, which is a surrogate marker for p65 activation, was decreased in the presence of HHV-6B U28 (Figures 1E, 1F, and S1C). These results suggest that HHV-6B U28 impairs activation of p65.

### HHV-6B U28 relocalizes p65 to the domain in the endoplasmic reticulum

**Figure 2:**
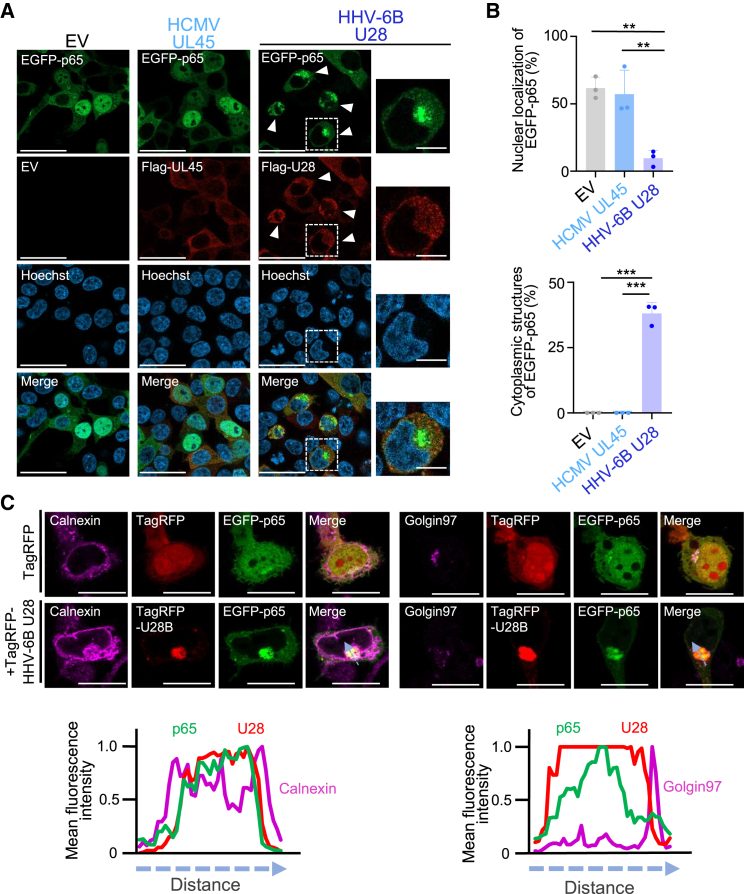
HHV-6B U28 relocalizes p65 to domains at the endoplasmic reticulum (A) HEK293T cells were co-transfected with EGFP-p65 and FLAG-HCMV UL45, FLAG-HHV-6B U28 or empty vector (EV). After 48 h, the cells were observed under a confocal microscope after staining with anti-FLAG antibody. Bars, 50 μm. Each image in the right-hand panels is the magnified image of the boxed area of the image in the left-hand panels (Bar, 10 μm). Arrowheads indicate the p65 structures. (B) Percentage of FLAG-RNR-expressing cells (50–100 cells in each experiment) showing nuclear localization of EGFP-p65 or cytoplasmic structures containing EGFP-p65 in experiments shown in (A). The data are means and standard deviations (*n* = 3; ∗∗, *p* < 0.01; ∗∗∗, *p* < 0.001 [Tukey’s test]). (C) Immunofluorescence microscopy images of HEK293T cells expressing EGFP-p65 and TagRFP-HHV-6B U28 or TagRFP, stained with the indicated antibodies. The images are representative of the results of the experiment. Bars, 20 μm. Fluorescence line scans along the dotted lines of fluorescence images are shown under each image. See also Figure S2.

Because HHV-6B U28 impairs phosphorylation of p65, we investigated whether HHV-6B U28 inhibits nuclear translocation of p65. HEK293T cells were transfected with plasmids expressing FLAG-HCMV UL45 or FLAG-HHV-6B U28 coupled with EGFP-p65 and analyzed by immunofluorescence. As shown in Figures 2A and 2B, EGFP-p65 was localized to the nucleus in 50% of the cells expressing EGFP-p65 when they were co-transfected with empty plasmid, even though they were not stimulated. The localization of EGFP-p65 might depend on its expression level, which varies in each cell. However, nuclear translocation of EGFP-p65 was strongly impaired in the presence of FLAG-HHV-6B U28 but not FLAG-HCMV UL45 (Figures 2A and 2B). In addition, 30–40% of cells expressing FLAG-U28 to block nuclear translocation of EGFP-p65 exhibited a structure in which EGFP-p65 was accumulated in the cytoplasm. Such structures were not present in cells expressing EGFP-p65 and FLAG-HCMV UL45. These observations suggest that HHV-6B U28 relocalizes p65 to a domain in the cytoplasm and thereby inhibits its translocation into the nucleus.

As shown in Figure 1F, HHV-6B U28 reduced phosphorylation of p65 Ser-536. To investigate the role of this phosphorylation, we analyzed the effect of HHV-6B U28 on p65 harboring an alanine mutation at Ser-536 (p65-S536A). EGFP-p65-S536A accumulated in the cytoplasm in the presence of HHV-6B U28, similar to the wild-type p65 (Figures S2A and S2B) and NF-κB activity induced by EGFP-p65 and EGFP-p65-S536A was blocked in the presence of HHV-6 U28 to a similar extent (Figure S2C). Thus, inhibition of phosphorylation of p65 at Ser-536 is not the reason for its accumulation in the cytoplasm, but this is rather the result of HHV-6B U28-mediated impairment of p65. Of note, TagRFP-conjugated HHV-6B U28 (TagRFP-HHV-6B U28) also exhibited relocalization of EGFP-p65 and EGFP-p65-S536A in a manner similar to that observed in FLAG-HHV-6B U28 (Figure S2A). However, the distribution of TagRFP-HHV-6B-U28 was more concentrated than that of FLAG-HHV-6B U28. It is possible that some of the domains that accumulated both EGFP-p65 and FLAG-HHV-6B U28 were hard to be stained by FLAG-antibody, whereas signals of the domains containing TagRFP-HHV-6B U28 were much stronger than those in the cytoplasm.

To further analyze the precise localization of structures of p65 in the presence of U28, HEK293T cells expressing EGFP-p65 and TagRFP-HHV-6B U28 were stained with organelle markers. This showed that the domains containing EGFP-p65 and TagRFP-HHV-6B U28 were partially colocalized with Calnexin but not with Golgin97 (Figure 2C). Of note, all sides of the domain were surrounded by Calnexin. Thus, the domain produced by HHV-6B U28 and p65 localized within the endoplasmic reticulum.

We used fluorescence recovery after photobleaching (FRAP) to test whether the HHV-6B U28-induced EGFP-p65 domain is immobilized in aggregates^19^^,^^20^ similar to MCMV M45. HEK293T cells were transfected with an EGFP-p65 plasmid coupled with TagRFP-HHV-6B U28 and observed by live-cell imaging. EGFP-p65 domains were bleached and fluorescence recovery was recorded for 420 s (Figures S2D and S2E). EGFP-p65 did not show any fluorescence recovery after photo bleaching for the duration of the experiment. By contrast, the fluorescence of EGFP-tagged NBR1 (negative control) recovered within a few minutes after bleaching, consistent with the liquid-like nature of NBR1.^21^ These results suggest that the cytoplasmic domain of HHV-6B U28 and p65 is aggregated.

### HHV-6B U28 specifically interacts with p65 and causes its relocalization, resulting in inhibition of NF-κB activation

In the canonical NF-κB pathway, p65 is regulated by the IKK complex consisting of IKKα, IKKβ, and NEMO, which is activated by RIPK1 (Figure 1A). As HHV-6B U28 induces relocalization of p65, we tested whether HHV-6B U28 effects the subcellular localization of RIPK1 or NEMO. HEK293T cells were transfected with the indicated plasmids and analyzed by immunofluorescence 48 h thereafter. HA-RIPK1 or EGFP-RIPK1 was localized in the punctate structures in the cytoplasm of 40% of cells when the empty plasmid was co-transfected. The structures of HA-RIPK1 or EGFP-RIPK1 were also observed in HHV-6B U28-expressing cells with no changes in their frequencies (Figures S3A and S3B). Interestingly, HHV-6B U28 accumulated in the structures of HA-RIPK1 or EGFP-RIPK1. Thus, HHV-6B U28 potentially interacts with RIPK1 but does not induce relocalization of RIPK1. Similarly, we did not observe any changes in the localization of NEMO in the presence of FLAG-HHV-6B U28 (Figure S3C). These observations suggest that HHV-6B U28 specifically relocalizes p65, the most downstream component of the canonical NF-κB pathway.

**Figure 3:**
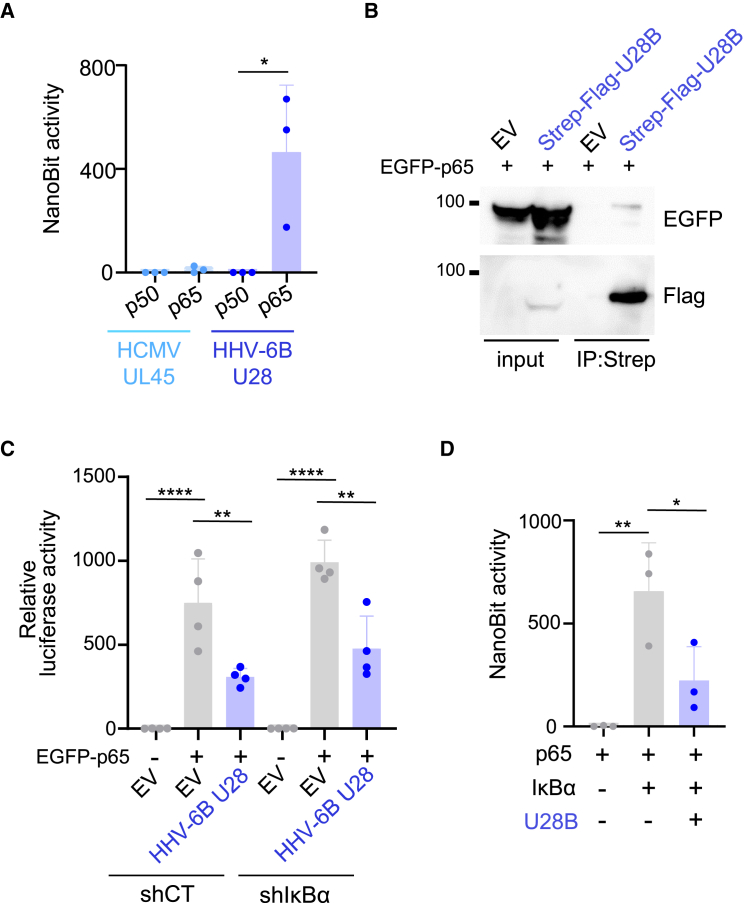
HHV-6B U28 interacts with p65 (A) HEK293T cells were co-transfected with the indicated combination of NanoBiT plasmids (LgBiT-p50 or p65 and SmBiT-HCMV UL45 or HHV-6B U28). After 24 h, luminescence was detected. Each value is shown after subtraction of the empty plasmid. Data are means and standard deviations. (*n* = 3; ∗, *p* < 0.05 [Two-tailed unpaired Student’s t test]). (B) HEK293T cells were transfected with Strep-FLAG-HHV-6B U28 expression plasmid or empty vector (EV). After 48 h the cells were lysed, and the extracts were subjected to precipitation using Strep-Tactin beads, followed by immunoblotting. (C) HEK293T-shCT or -shIκBα cells were co-transfected with the NF-κB-luc reporter plasmid, pRL-TK and the indicated expression plasmids. The luciferase activity was measured 24 h post-transfection. The data are shown as means and standard deviations (*n* = 4; ∗∗, *p* < 0.01; ∗∗∗∗, *p* < 0.0001 [Tukey’s test]). (D) HEK293T cells were co-transfected with the indicated combination of NanoBiT plasmids (LgBiT-p65 and SmBiT-IκBα) and with or without TagRFP-HHV-6B U28. Total plasmid volume was normalized with empty plasmid. After 24 h, luminescence was detected. Each value is shown after subtraction of the empty plasmid. The data are shown as means and standard deviations (*n* = 3; ∗, *p* < 0.05; ∗∗, *p* < 0.01 [Tukey’s test]). See also Figure S2.

Next, interactions between HHV-6B U28 and p65 were analyzed by the NanoBiT assay, which enables measurement of protein-protein interactions in living cells. In this assay, the luciferase is split into two subunits, LgBiT and SmBiT, which do not luminesce when separated, but luciferase activity is reconstituted when they are in close proximity. Co-expression of LgBiT-p65 and SmBiT-HHV-6B U28 significantly increased luminescence, whereas LgBiT-p50 and SmBiT-HHV-6B U28 did not increase luminescence (Figure 3A). In contrast, SmBiT-HCMV UL45 did not enhance luminescence in the presence of LgBiT-p65. EGFP-p65 was co-precipitated with Strep-tagged HHV-6B U28 from the lysate of HEK293T cells (Figure 3B).

As IκBα inhibits NF-κB activation by binding to p65 (Figure 1A), we analyzed the significance of IκBα in HHV-6B U28-mediated NF-κB inhibition. HEK293T cells expressing short hairpin (sh)RNA to IκBα produced less IκBα than those expressing control shRNA (Figure S4A). These cells were then co-transfected with a plasmid expressing FLAG-HHV-6B U28 or control plasmid coupled to NF-κB-luc and EGFP-p65, and luciferase assays were performed. As expected, the cells expressing shRNA to IκBα exhibited increased NF-κB activity compared to the control cells in all independent experiments, and HHV-6B U28 expression impaired this activity in both cells (Figure 3C), indicating that HHV-6B abrogates NF-κB activity independent of IκBα. As RIPK1 and NEMO activate p65 through IκBα degradation, these results suggest that HHV-6B U28 impairs NF-κB activation through its interaction with p65.

To reveal the effects of HHV-6B U28 in the NF-κB cascade, we analyzed interactions between p65 and IκBα in the presence of HHV-6B U28, as detected by NanoBiT assay (Figure 3D). The p65-IκBα interaction was found to be abrogated in the presence of HHV-6B U28. We also analyzed the subcellular localization of IκBα in the presence of HHV-6B U28. HEK293T cells were transfected with a plasmid expressing TagRFP-HHV-6B U28 together with EGFP-p65, followed by immunofluorescence analysis using an anti-IκBα antibody. As shown in Figure S4B, IκBα was not detected in the structure in which HHV-6B U28 and p65 were colocalized. These results suggest that HHV-6B U28 impairs p65-IκBα interactions, thereby resulting in the accumulation of p65 in the cytoplasm.

**Figure 4:**
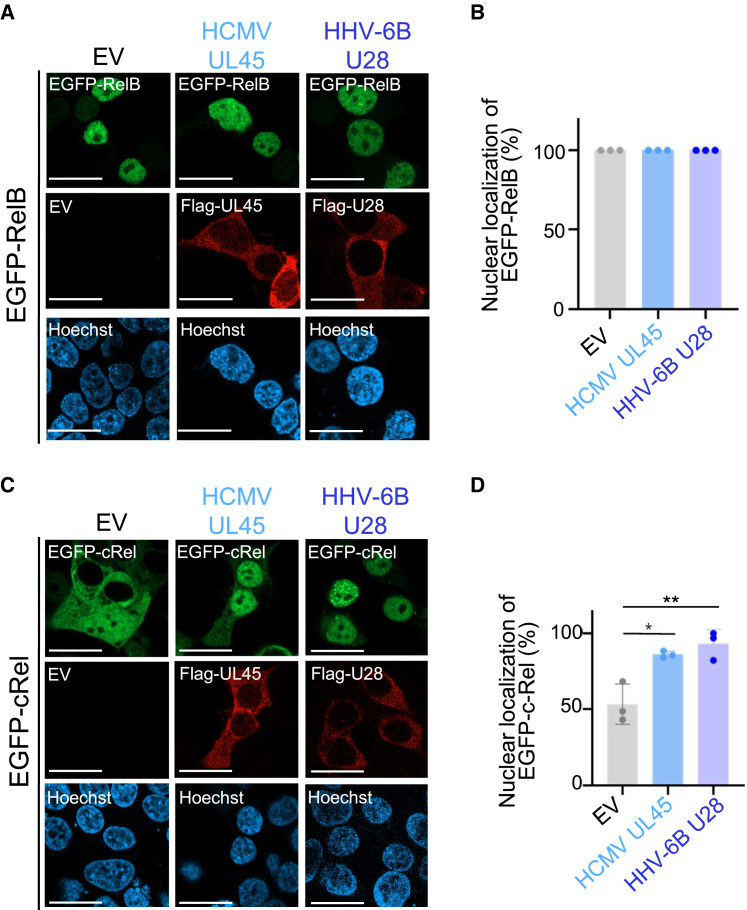
HHV-6B U28 does not relocalize RelB and c-Rel (A and C) HEK293T cells were co-transfected with EGFP-RelB (A) or EGFP-*c*-Rel (C) together with FLAG-HCMV UL45, FLAG-HHV-6B U28 or empty vector (EV). After 48 h, the cells were observed under a confocal microscope after staining with anti-FLAG antibody. Bars, 20 μm. (B and D) The percentage of FLAG-RNR-expressing cells (50–100 cells in each experiment) showing nuclear localization of EGFP-RelB (B) or EGFP-c-Rel (D) in experiments from (A) or (C). The data are shown as means and standard deviations (*n* = 3; ∗, *p* < 0.05; ∗∗, *p* < 0.01 [Tukey’s test]). See also Figure S3.

In addition to the canonical NF-κB cascade, an alternative non-canonical cascade exists in cells.^4^ In this non-canonical NF-κB cascade, RelB instead of p65 is activated through inducible processing of NF-κB p100. In the canonical NF-κB cascade, the other NF-κB subunit c-Rel can also function instead of p65. Inhibition of p65 by HHV-6B U28 is specific, because HHV-6B U28 expression did not inhibit nuclear translocation of RelB or c-Rel (Figure 4). Rather, HHV-6B U28 and HCMV UL45 enhanced nuclear translocation of c-Rel. Taken together, these results suggest that HHV-6B U28 specifically interacts with p65 and sequesters it in the cytoplasm, resulting in inhibition of NF-κB activation.

### The N-terminal region of HHV-6B U28 is important for p65 sequestration

**Figure 5:**
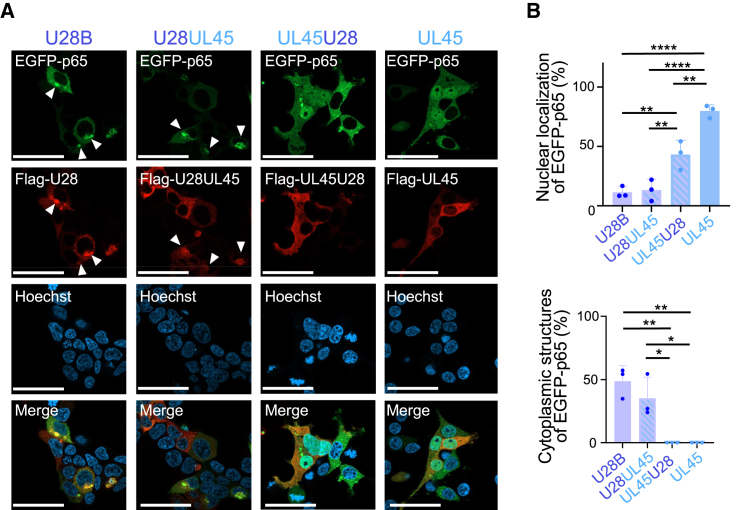
The N-terminal region of HHV-6B U28 is important for p65 sequestration (A) Fluorescence microscopy images of HEK293T cells expressing EGFP-p65 and the indicated RNR chimera. Arrowheads indicate the cytoplasmic structures with p65. Bars, 50 μm. (B) The percentage of FLAG-RNR-expressing cells (50–100 cells in each experiment) showing nuclear localization of EGFP-p65 or cytoplasmic structures containing FLAG-RNR and EGFP-p65 in experiments from (A). The data are shown as means and standard deviations (*n* = 3; ∗, *p* < 0.05; ∗∗, *p* < 0.01; ∗∗∗∗, *p* < 0.0001 [Tukey’s test]). See also Figure S6.

As nuclear translocation of p65 was inhibited by HHV-6B U28 but not by HCMV UL45 (Figure 2A), we constructed plasmids expressing U28 and UL45 chimeras conjugated with FLAG tag to determine the region in HHV-6B U28 responsible for inhibiting p65. HEK293T cells were transfected with these plasmids together with EGFP-p65 and assayed by immunofluorescence. A chimeric protein containing the N-terminal half of HHV-6B U28 and the C-terminal half of HCMV UL45 (U28UL45) inhibited the nuclear translocation of EGFP-p65, and formed structures in the cytoplasm at similar percentages to the wild-type U28 (Figures 5A and 5B). A chimera with the N-terminal half of HCMV UL45 and the C-terminal half of HHV-6B U28 (UL45U28) inhibited nuclear translocation of EGFP-p65 but with a lesser effect than U28UL45 and without producing cytoplasmic structures with EGFP-p65. This observation suggests that the N-terminal region of HHV-6B U28 is important for the sequestration of p65.

### U28 inhibits p65 activation and suppresses cytokine production during HHV-6B infection

Because phosphorylation of p65 was decreased by exogenous expression of HHV-6B U28 (Figures 1E and 1F), we investigated whether phosphorylated p65 was also reduced in HHV-6B-infected cells. MT4 cells were mock-infected or infected with HHV-6B HST for 48 h and analyzed by immunoblotting. As shown in Figures S5A and S5B, the ratio of phosphorylated p65/total p65 in HHV-6B-infected cells was slightly but significantly reduced relative to mock-infected cells 48 h after infection.

**Figure 6:**
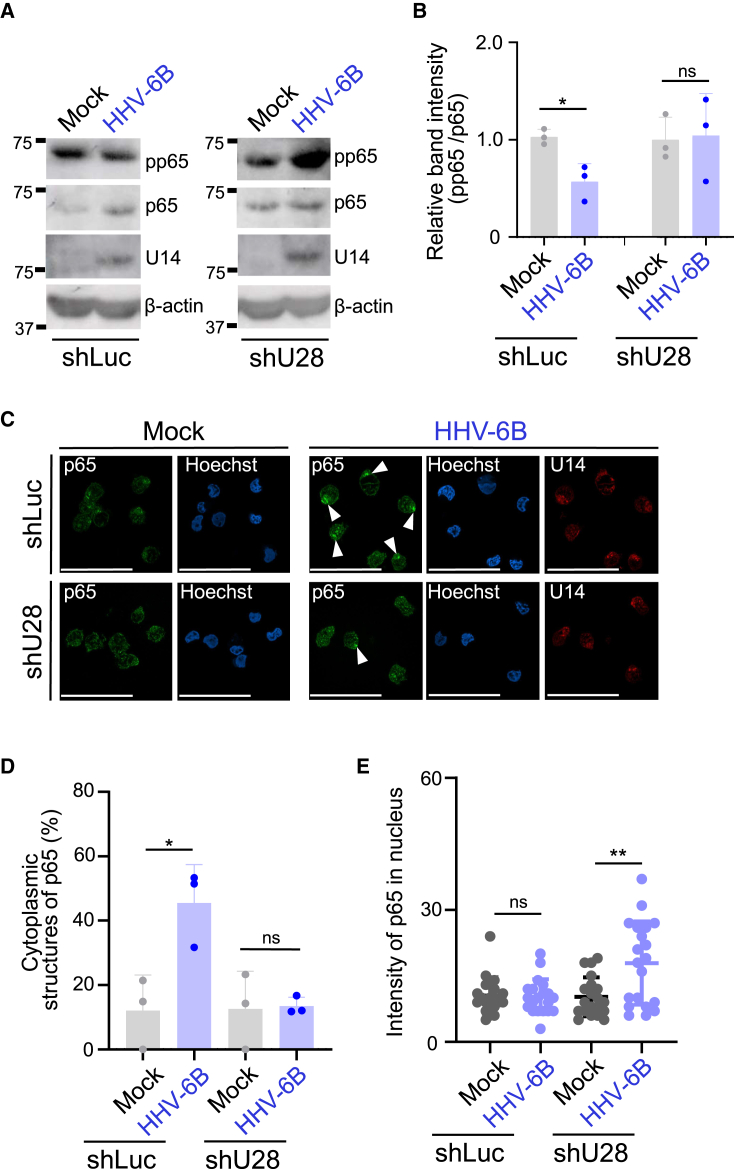
U28 inhibits p65 activation during HHV-6B infection (A) 4 × 10^5^ MT4-shLuc or MT4-shU28 cells were mock-infected or infected with HHV-6B HST for 48 h and analyzed by immunoblotting. (B) The intensities of pp65 in each lane in (A) normalized to p65 expression. The data are shown as means and standard deviations (*n* = 3; ∗, *p* < 0.05 [Welch’s t test]). (C) 1 × 10^6^ MT4-shLuc or MT4-shU28 cells were mock-infected or infected with HHV-6B HST for 96 h and analyzed with immunofluorescence. Bars, 50 μm. Images are representative of 3 independent experiments. (D) The percentage of cytoplasmic structures with p65 in cells (50–100 cells in each experiment) in experiments from (C). The data are shown as means and standard deviations (*n* = 3; ∗, *p* < 0.05 [Welch’s t test]). (E) The fluorescence intensity of p65 (*n* = 20) in the nucleus was quantified. Data are representative of three independent experiments. The data are shown as means and standard deviations (*n* = 20; ∗∗, *p* < 0.01 [Welch’s t test)]). See also Figure S5.

Next, we investigated the importance of HHV-6B U28 in HHV-6B-infected cells. MT4 cells were transduced with lentiviral vector expressing shRNA targeted to U28 (MT4-shU28) or luciferase (MT4-shLuc) as a control. As expected, expression of U28 shRNA significantly reduced the amount of U28 mRNA in infected cells (Figure S5C). To analyze the effect of shRNA to U28 on viral replication, MT4-shLuc or MT4-shU28 cells were infected with HHV-6B HST. These cells were then incubated for 6 days to allow multiple replication cycles, followed by DNA extraction. Viral genome copy number in the MT4-shU28 cells was identical to MT4-shLuc cells (Figure S5D). As expected, reduction of the amount of phosphorylated p65 in infected MT4-shLuc cells was prevented in infected MT4-shU28 cells (Figures 6A and 6B). Viral protein U14 was detected to the same extent in both cells, suggesting that these cells were infected with HHV-6B and produced viral proteins properly in MT4-shU28 cells.

Next, we analyzed the subcellular localization of p65 in infected cells. MT4-shLuc or MT4-shU28 cells were mock-infected or infected with HHV-6B. After 72 h, the cells were analyzed by immunofluorescence. In accordance with experiments using HEK293T cells expressing HHV-6B U28, HHV-6B-infected MT4-shLuc cells produced a structure in which p65 accumulated in the cytoplasm (Figures 6C and 6D). These domains of p65 in infected cells were rarely observed in MT4-shU28 cells infected with HHV-6B, suggesting that U28 is responsible for this p65 relocalization. Furthermore, p65 was localized in the nucleus in a major fraction of infected MT4-shU28 cells, whereas it was mainly localized in the cytoplasm in the infected MT4-shLuc cells (Figure 6E). This indicates that viral infection enhances nuclear localization of p65 but U28 impairs this translocation.

**Figure 7:**
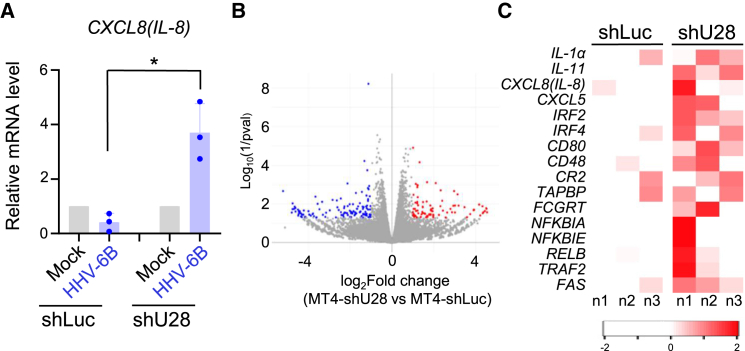
U28 suppresses NF-κB-dependent cytokine expression during HHV-6B infection (A) 2 × 10^5^ MT4-shLuc or MT4-shU28 cells were mock-infected or infected with HHV-6B HST. At 48 h post-infection, the cells were incubated with TNF-α (20 ng/mL) for 10 min and the mRNA was collected from these cells. The expression of mRNA for CXCL8 (*IL-8)* was quantified by RT-qPCR. Relative amounts of these mRNAs were normalized to β-actin. The data are shown as means and standard deviations (*n* = 3; ∗, *p* < 0.05 [Welch’s t test]). (B) Volcano plot of the differentially expressed genes (DEGs). Genes from shU28-up signature (red) and shU28-down signature (blue) are highlighted. (C) Heatmap of selected DEGs between MT4-shLuc or shU28. Representative NF-κB target genes are provided. Normalized *Z* score values were calculated for each differentially expressed gene (high, red; low, white). The distribution of the level of expression of the DEGs is shown on the color key legend (below).

Next, we analyzed whether U28 inhibits cytokine production stimulated by TNF-α. MT4-shLuc or MT4-shU28 cells were mock-infected or infected with HHV-6B and incubated with TNF-α for 48 h. The amount of *CXCL8 (IL-8)* mRNA in HHV-6B-infected MT4-shLuc cells was significantly higher than in HHV-6B-infected MT4-shU28 cells (Figure 7A), suggesting that U28 suppresses TNF-α-dependent cytokine production during HHV-6B infection.

Because the transcription factor NF-κB regulates multiple aspects of immune function and serves as a pivotal mediator of inflammatory responses,^4^ we further analyzed the genes regulated by HHV-6B U28 using RNA-seq. MT4-shLuc or shU28 cells were infected with HHV-6B and RNA extracted 72 h thereafter. We then conducted a global RNA sequencing analysis of changes in whole genome expression in MT4-shLuc versus MT4-shU28 cells (Figure 7B). As shown in Figure 7C, inflammatory cytokines and chemokines, such as *IL-1α*, *IL-11*, and *IL-8*, which are known to be regulated by NF-κB, were induced in the absence of U28 in infected cells. Taken together, these results suggest that HHV-6B U28 blocks activation of NF-κB to repress antiviral responses.

## Discussion

Viral ribonucleotide reductases are critical for the replication of alpha- or gammaherpesviruses *in vivo*,^22^^,^^23^ but it has remained unclear why enzymatically inactive RNR R1s are conserved in betaherpesviruses. Here, we showed that inhibition of NF-κB signaling is a conserved function of RNR R1 in *Betaherpesvirinae* roseoloviruses. Like the HCMV RNR R1, UL45, the HHV-6B RNR R1 encoded by the U28 gene led to lower expression of inflammatory cytokines. However, the precise mechanism responsible for the inhibition of NF-κB signaling is different in different betaherpesviruses, namely, HCMV UL45 targets RIPK1, whereas MCMV M45 impairs RIPK1, RIPK3, and NEMO.^9^^,^^17^^,^^18^^,^^20^ In the present study, we showed that HHV-6B U28 interacts with p65, represses phosphorylation at Ser-536 and blocks its translocation into the nucleus. HHV-6B U28 possibly interacts with RIPK1 but it is not required for the HHV-6B U28-mediated inhibition of NF-κB activation.

NF-κB is a master regulator of antiviral immune responses and viruses have evolved different mechanisms to counteract this.^7^ It is a common ploy for viruses to encode multiple proteins that inhibit NF-κB in different ways. In addition to the role of HCMV UL45, several HCMV proteins have been reported to inhibit NF-κB signaling. For example, UL83 blocks the DNA sensor IFI16, UL26 inhibits IKK proteins, and HCMV UL48 cleaves polyubiquitin chains of RIPK1 through its deubiquitinating enzyme activity.^17^^,^^24^^,^^25^ Hence, the contribution of each individual viral protein may be minor in infected cells; thus, it has been reported that deletion of HCMV UL45 only slightly reduces progeny viral yields.^16^^,^^17^ We could not observe a significant reduction of viral yields in MT4-shU28 cells (Figure S5D). In this context, the RNR R1s of them possibly cooperate with other viral proteins to enhance viral replication. One of the candidates would be HCMV UL48 homologues, as HCMV UL45 cooperates with UL48 to suppress RIPK1-mediated NF-κB signaling.^17^

Furthermore, some of the viral components activate NF-κB signaling during viral attachment and after envelope-membrane fusion to enhance viral gene expression.^26^^,^^27^^,^^28^^,^^29^^,^^30^^,^^31^ As NF-κB is critical for cell survival and proliferation in addition to its antiviral function,^5^ viruses hijack NF-κB-dependent signaling cascades and utilize them for their replication. Thus, the functional interaction between NF-κB and herpesvirus infection is complex and must be tightly regulated. Viral RNRs are categorized as early proteins important for viral genome replication.^17^^,^^30^^,^^32^^,^^33^ Our results, together with the report on HCMV UL45^17^ suggest that it is important to inhibit NF-κB signaling and host antiviral response during or after viral genome replication of betaherpesviruses.

Similar to HHV-6B U28, some of the herpesviral proteins were reported to inhibit p65 directly. HSV-1 Us3 induces hyperphosphorylation of p65 at Ser-75 and blocks its nuclear translocation.^34^ HSV-1 UL24, UL42, and VP16 bind p65 to inhibit its activation.^35^^,^^36^^,^^37^ KSHV LANA-1 induces ubiquitination and degradation of p65.^38^ Although these mechanisms of inhibition are different, p65 is commonly targeted by these different viruses. HHV-6B U28 inhibited nuclear translocation of p65 but not the non-canonical NF-κB component RelB or c-Rel (Figure 4). Rather, HHV-6B U28 enhanced nuclear translocation of c-Rel via an unidentified mechanism, as does HCMV UL45. The c-Rel transcription factor has DNA-binding residues that are distinct from other NF-κB proteins, and plays an important role in lymphocyte differentiation and function.^39^^,^^40^ Thus, enhancement of nuclear translocation of c-Rel by HHV-6B U28 might contribute to viral replication. Further studies are required to establish the exact role of c-Rel in infected cells.

In this manuscript, we describe the importance of the residues at the N-terminal region (1–400) of HHV-6B U28 for inhibition of NF-κB signaling. Replacement of the residues 1–400 of HHV-6B U28 with HCMV UL45 resulted in a loss of inhibitory effects on p65. The residues in the external domain of the N-terminal extension of HHV-6B U28 are highly conserved in the roseoloviruses U28 but not in the HCMV UL45. Thus, HHV-6B U28 possibly binds p65 through this region (Figure S6). It has been reported that MCMV M45 and HSV-1 RNR R1 (UL39) interact with RIPK1 and RIPK3 through their RIP homotypic interaction motif (RHIM) domain at the N-terminal region and induced protein aggregation motif (IPAM) at the C-terminal region.^9^^,^^20^ However, the region containing RHIM as well as the core residues of IPAM are not conserved in roseolovirus U28 (Figure S6). Future work on fine mapping of the regions in HHV-6B U28 responsible for these interactions will be required to understand the molecular mechanisms responsible for the character of roseolovirus U28.

Various different stimuli including pathogen-associated molecular patterns (PAMPs) activate NF-κB through multiple pathways. RIPK1 is critical for NF-κB activation upon TNFR1 and TLR3 but not TLR2, TLR4 or interleukin 1 receptor ligation.^4^ Despite this, all these signaling pathways finally activate p65. The contribution of each pathway for NF-κB activation varies depending on cell type. As tropisms of cytomegaloviruses and roseoloviruses are completely different, they possibly exploit different individual target proteins to inhibit NF-κB depending on the cellular context. Our findings provide an example of herpesvirus immune evasion machinery, which is fine-tuned for each viral species.

### Limitations of the study

This study documented a role of HHV-6B U28 for inhibiting NF-κB signaling. However, we did not document direct interactions or show structural analyses for roseolovirus U28 and p65. Biochemical analysis will reveal the mechanisms responsible for this inhibition. To analyze the role of U28 in infected cells, we used cells expressing shRNA to U28, but we could not completely abolish expression of this gene and could not use U28 knock-out virus. Thus, the effect of U28 in infected cells could not be unequivocally evaluated in this study. Furthermore, elucidation of the involvement of U28 in the pathogenesis of HHV-6B in terms of drug development remains challenging. Further studies on cocultures of infected cells and effector cells or using humanized mice will be necessary to define the precise role of HHV-6B U28 for the immune response and HHV-6B pathogenesis.

## Resource availability

### Lead contact

Further information and requests for resources and reagents should be directed to and will be fulfilled by the lead contact, Jun Arii (jarii@med.kobe-u.ac.jp).

### Materials availability

This study did not generate new unique reagents. All the materials generated in this study are available from the lead contact upon request.

### Data and code availability

• RNA-seq data have been deposited at DDBJ and are publicly available.
• This paper does not report the original code.
• Any additional information required to reanalyze the data reported in this paper is available from the lead contact upon request.

## Acknowledgments

We thank Yasuyo Ueda and Mitsuhiro Nishimura for their excellent technical assistance, J. Schmid, J. Song, K. Guan, J. Debnath, W. Brune, K. Mostov, S. Dias and S. Elledge for kindly providing reagents, and lab members for helpful comments. RNA-seq analysis was supported by Rhelixa, Inc. This study was supported by grants for Scientific Research from the Japan Society for the Promotion of Science (JSPS), the Ministry of Education, Culture, Science, Sports and Technology of Japan (10.13039/501100001700MEXT) Leading Initiative for Excellent Young Researchers Grant, 10.13039/501100025019SPRING (JPMJSP2148) from 10.13039/501100002241Japan Science and Technology Agency, contract research funds from the Japan Program for Infectious Diseases Research and Infrastructure (20wm0325005h) and Precursory Research for Innovative Medical Care (PRIME, 22gm6410022h) from the 10.13039/100009619Japan Agency for Medical Research and Development and grants from the 10.13039/100007449Takeda Science Foundation, MSD Life Science Foundation, Shionogi Infectious Disease Research Promotion Foundation and the Waksman Foundation of Japan. K. Amaliin and S. Aktar was supported by a research fellowship from 10.13039/501100001700MEXT.

## Author contributions

Conceptualization: M.H. and J.A.; methodology: M.H., K.A., J.R.H., and S.A. investigation: M.H., K.A., and J.A.; writing: M.H. and J.A.; supervision: Y.M. and J.A.

## Declaration of interests

The authors declare no competing interests.

## STAR★Methods

### Key resources table

**Table:** 

REAGENT or RESOURCE	SOURCE	IDENTIFIER
**Antibodies**		

Mouse monoclonal anti-Flag (M2)	Sigma-Aldrich	Cat# F3165, RRID:AB_259529
Mouse monoclonal anti-β-actin (AC15)	Sigma-Aldrich	Cat# ZRB1312, RRID:AB_3083534
Mouse monoclonal anti-α-tubulin (DM1A)	Sigma-Aldrich	Cat# MABT205, RRID:AB_11204167
Rabbit monoclonal anti-p-p65 (Ser536) (93H1)	Cell Signaling Technology	Cat# 3031, RRID:AB_330559
Rabbit monoclonal anti-p65 (D14E12)	Cell Signaling Technology	Cat# 8242, RRID:AB_10859369
Mouse monoclonal anti-p65 (L8F6)	Cell Signaling Technology	Cat# 6956, RRID:AB_10828935
Mouse monoclonal anti-IκBα (L35A5)	Cell Signaling Technology	Cat# 4814, RRID:AB_390781
Mouse monoclonal anti-NEMO (DA10-1)	Cell Signaling Technology	Cat# 2695, RRID:AB_2124826
Rabbit polyclonal anti-Calnexin (ab22595)	Abcam	Cat# ab22595, RRID:AB_2069006
Mouse monoclonal anti-Golgin-97 (CDF4)	Thermo Fisher Scientific	Cat# PA5-30048, RRID:AB_2547522
Mouse monoclonal anti-HA (TANA2)	MBL International	Cat# M180-7, RRID:AB_11124961
Rabbit polyclonal anti-GFP	MBL International	Cat# 598, RRID:AB_591819
Mouse monoclonal anti-HHV-6B gB (OHV-1)	Okuno et al.,^41^	N/A
Rabbit polyclonal anti-HHV-6B IE1	Mori et al.,^42^	N/A
Rabbit polyclonal anti-HHV-6B U14	Takemoto et al.,^43^	N/A
Anti-rabbit IgG, HRP-linked Antibody	Cell Signaling Technology	Cat #7074, RRID:AB_2099233
Anti-mouse IgG, HRP-linked Antibody	Cell Signaling Technology	Cat #7076, RRID:AB_330924
Goat anti-Mouse IgG (H+L) Highly Cross-Adsorbed Secondary Antibody, Alexa Fluor Plus 488	Thermo Fisher Scientific	Cat # A32731, RRID:AB_2633280
Goat anti-Rabbit IgG (H+L) Highly Cross-Adsorbed Secondary Antibody, Alexa Fluor Plus 488	Thermo Fisher Scientific	Cat# A11034 RRID:AB_2576217
Goat anti-Mouse IgG (H+L) Highly Cross-Adsorbed Secondary Antibody, Alexa Fluor Plus 594	Thermo Fisher Scientific	Cat # A32742, RRID:AB_2762825
Goat anti-Rabbit IgG (H+L) Highly Cross-Adsorbed Secondary Antibody, Alexa Fluor Plus 594	Thermo Fisher Scientific	Cat # A32740, RRID:AB_2762824
Goat anti-Mouse IgG (H+L) Highly Cross-Adsorbed Secondary Antibody, Alexa Fluor Plus 647	Thermo Fisher Scientific	Cat # A32728, RRID:AB_2633277
Goat anti-Rabbit IgG (H+L) Highly Cross-Adsorbed Secondary Antibody, Alexa Fluor Plus 647	Thermo Fisher Scientific	Cat # A32733, RRID:AB_2633282

**Bacterial and virus strains**		

HHV-6B strain HST	Tang et al.,^43^	N/A

**Chemicals, peptides, and recombinant proteins**		

DMEM	Shimadzu	Cat # 5919
RPMI	Sigma-Aldrich	Cat # R8758
FBS	Thermo Fisher Scientific	Cat # 10270-106
Doxycycline	TaKaRa Bio	Cat # 631311
Puromycin	Invivogen	Cat # ant-pr-1
Blasticidin	Fujifilm	Cat # 029-18701
TNF-α	PeproTech	Cat # 300-01A
Protease inhibitor cocktail	Nacalai Tesque	Cat # 25955
MagStrep “type3” Strep-Tactin beads	IBA	Cat # 2-1613-002

**Critical commercial assays**		

Dual-Luciferase Reporter Assay System	Promega	Cat # E1910
Nano-Glo Live Cell Reagent	Promega	Cat # N2012
NucleoSpin RNA kit	MachereyNagel	Cat # 740955
SuperScript IV First-Strand Synthesis System	Thermo Fisher Scientific	Cat # 18091050
SYBR Select master mix	Thermo Fisher Scientific	Cat # 4472919
DNeasy Blood & Tissue Kit	QIAGEN	Cat # 69504
Maxwell RSC simplyRNA cells kit	Promega	Cat # AS1390
NEBNext Poly(A) mRNA Magnetic Isolation Module	New England Biolabs	Cat # E7490L
NEBNext Ultra II Directional RNA Library Prep Kits	New England Biolabs	Cat # E7760L

**Deposited data**		

Bulk RNA-seq datasets of MT4-shLuc and MT4-shU28 cells infected with HHV-6B	DDBJ	PRJDB18205

**Experimental models: Cell lines**		

HEK293T cells	Y. Mori	N/A
MT4 cells	Y. Mori	N/A

**Experimental models: Organisms/strains**		

Umbilical cord blood mononuclear cells (CBMCs)	The Cell Bank of the RIKEN BioResource Center	N/A

**Oligonucleotides**		

Primers for RT-qPCR	see Table S1,^31^	N/A
Primers for Calculation of virus genome copy numbers	see Table S1,^31^	N/A
Oligonucleotides to construct shRNA expressing vectors	see Table S1	N/A

**Recombinant DNA**		

Plasmid: pcDNA3.1/myc-His A	Thermo Fisher Scientific	Cat # V80020
Plasmid: pCAG-OSF (One-Strep-Flag)	Morita et al.,^36^	N/A
Plasmid: pTagRFP-C	Evrogen	Cat # FP141
Plasmid: pEGFP-p65	Schmid et al.,^44^	Addgene plasmid #111190
Plasmid: pcDNA3-HA-RIPK1	Seo et al.,^45^	Addgene plasmid #78834
Plasmid: pcDNA3-HA-human NEMO	Tang et al.,^46^	Addgene plasmid #13512
Plasmid: pEGFP-C1	Clontech	Cat # 6084-1
Plasmid: pMXspuro-GFP-NBR1	Kenific et al.,^47^	Addgene plasmid #74202
Plasmid: pGL4.32 (NF-κB-luc)	Promega	Cat # E8491
Plasmid: pRL-SV40	Promega	Cat # E2231
Plasmid: pRL-TK	Promega	Cat # E2241
Plasmid: pTK-N-LgBiT	Promega	Cat # N2014
Plasmid: pTK-C-LgBiT	Promega	Cat # N2014
Plasmid: pTK-N-SmBiT	Promega	Cat # N2014
Plasmid: pInducer10	Meerbrey et al.,^48^	Addgene plasmid # 44011
Plasmid: pLKO.1-blast shGFP	Dias et al.	Addgene, Plasmid #110419
Plasmid: pLKO.1-blast	Bryant et al.,^49^	Addgene plasmid # 26655
Plasmid: pCAG-HIV-gp	Miyoshi et al.,^50^	N/A
Plasmid: pCMV-VSV-G-RSV-Rev	Miyoshi et al.,^50^	N/A

**Software and algorithms**		

Genetycs ver. 2.2.5	Nihon Server Corporation	https://www.genetyx.co.jp/
MEGA 11	Tamura et al.,^51^	https://www.megasoftware.net/
GraphPad Prism 7	GraphPad Software	
Heatmapper	Babicki et al.^52^	http://www.heatmapper.ca/
ZEN3.1	Zeiss	www.zeiss.com

**Other**		

Fusion FX6.EDGE	Vilber Bio Imaging	https://www.vilber.com/
LSM800 microscope	Zeiss	https://www.zeiss.com/microscopy/ja/home.html
Fluorescence microscope; BZ-X810	KEYENCE	https://www.keyence.co.jp/
QuantStudio 1	Thermo Fisher Scientific	https://www.thermofisher.com/jp/ja/home.html

### Experimental model and study participant details

#### Cells

HEK293T cells were maintained in Dulbecco’s modified Eagle’s medium (DMEM) medium containing 8% fetal bovine serum (FBS).^53^^,^^54^ Transfection experiments were performed using polyethylenimine for single plasmids and Lipofectamine 3000 (Thermo Fisher Scientific) for multiple plasmids. MT4 cells (T-cell line) were cultured in RPMI 1640 medium containing 8% FBS.^55^^,^^56^ Umbilical cord blood mononuclear cells (CBMCs) were purchased from the Cell Bank of the RIKEN BioResource Center, Tsukuba, Japan and were cultured as described previously.^31^^,^^55^ The use of CBMCs in this study was approved by the Ethics Committee of Kobe University Graduate School of Medicine (approval number: No.1209). The cell lines have not been authenticated or tested for mycoplasma contamination. Sample size calculation was not performed in this study.

#### Viruses

HHV-6B strain HST was propagated in activated CBMCs until the cytopathic effect was maximal.^57^ The cells were lysed by freezing and thawing once from -80°C and used as viral suspensions. Indicated numbers of MT4-shLuc or MT4-shU28 cells were mixed with virus suspensions (4-6 x 10^10^ genome copies/ml) and centrifuged at 35°C, 1700 rpm for 35 min, followed by culturing in RPMI with 1% FBS. At 48 h post-infection, the infected cells were analyzed by immunoblotting. Infection was confirmed using mAb against HHV-6B gB protein.

### Method details

#### Plasmids

Plasmids pcDNA3.1-Flag-HHV-6A U28, pcDNA3.1-Flag-HHV-6B U28 and pcDNA3.1-Flag-HHV-7 U28 used for expression of Flag-tagged and codon-optimized HHV-6A, HHV-6B or HHV-7 U28, respectively, were constructed by Thermo Fisher Scientific. Plasmid Strep-Flag-HHV-6B U28 used for affinity precipitation was constructed by cloning the open reading frame (ORF) of HHV-6B U28 into pCAG-OSF (One-Strep-Flag) plasmid.^58^ Plasmids pcDNA3-Flag-HCMV UL45 used for expression of Flag-tagged HCMV UL45 were constructed by cloning the ORF of HCMV UL45, amplified by PCR from the genome of HCMV Merline strain into pcDNA3. The TagRFP sequence was amplified by PCR from pTagRFP-C (Evrogen) and cloned into pcDNA3.1-Flag-HHV-6B U28 to produce pTagRFP-HHV-6B U28 to express a fusion protein of TagRFP and HHV-6B U28. Plasmid pEGFP-p65^44^ was used to express an EGFP-p65 fusion protein. To construct EGFP-p65 S536A, PCR-based site mutagenesis was performed as previously described.^53^^,^^59^ Plasmid pcDNA3-HA-RIPK1^45^ and pcDNA3-HA-human NEMO^46^ was used to express HA-RIPK1 or HA-NEMO, respectively. Plasmid pEGFP-RelB and pEGFP-c-Rel were constructed by PCR amplification of the RelB or c-Rel sequence from the cDNA synthesized from the total RNA of JJhan or HEK293T cells, respectively and cloned into pEGFP-C1 (Clontech). Plasmid pMXspuro-GFP-NBR1^47^ was used to express EGFP-NBR1.

Plasmids pcDNA3-Flag-U28BUL45 and -UL45U28B, carrying the fusion proteins of the HHV-6B U28-HCMV UL45 chimeras with Flag tag, were constructed by amplification from plasmids pcDNA3.1-Flag-HHV-6B U28 or pcDNA3-Flag-HCMV UL45 and cloning into pcDNA3. U28BUL45 or UL45U28B are chimeras of HHV-6B U28 (codons 1 to 400) and HCMV UL45 (codons 556-1181) or HCMV UL45 (codons 1 to 555) and HHV-6B U28 (codons 401-804), respectively.

For NanoBiT assays, the coding sequences of p65 or IκBα were amplified by PCR from the pEGFP-p65 or cDNA synthesized from the HEK293T RNA and cloned into pTK-N-LgBiT (Promega) or pTK-N-SmBiT (Promega), respectively. They are designated pLgBiT-p65 or pSmBiT-IκBα, respectively. To construct pLgBiT-p50 and pSmBiT-p50, the coding sequences of p50 were amplified by PCR from the cDNA synthesized from HEK293T RNA. The coding sequence of SmBiT was amplified by PCR from pTK-N-SmBiT and cloned into pTagRFP-HHV-6B U28 to produce pSmBiT-HHV-6B U28. To construct pSmBiT-UL45, the coding sequences of UL45 was amplified by PCR from the pcDNA3-Flag-HCMV UL45 cloned into pTK-N-SmBiT.

pInd10-Luc and pInd10-U28, lentivirus vectors expressing shRNA targeting Luciferase or U28, respectively, under the control of a Doxycycline-inducible promoter, were constructed by PCR amplification of the shLuc or shU28 oligo sequence with pInd10-F and pInd10-R primers (Table S1), and cloned into pInducer10.^48^

To produce pLKO-IκBα, sense and antisense oligonucleotides^60^ (Table S1) were designed for insertion into the *Age*I and *EcoRI* site in the pLKO.1-blast shRNA expression vector.^49^ pLKO.1-blast shGFP (Addgene, Plasmid #110419) was used for control.

#### Construction of cell lines

HEK293T cells were transfected with pInd10-Luc or pInd-U28 along with packaging plasmids pCAG-HIV-gp and pCMV-VSV-G-RSV-Rev^50^ as previously described.^55^^,^^61^^,^^62^ At 48 h post-transfection, the supernatants of the transfected cells were harvested, and MT4 cells were transduced with these supernatants and selected with 1 μg/ml of puromycin. Resistant cells were designated MT4-shLuc or MT4-shU28, respectively. For induction of shRNA to U28, MT4-shLuc or MT4-shU28 cells were treated with 20 μg/ml Doxycycline (Dox) for 24 h before infection. Similarly, HEK293T-shGFP or -shIκBα was constructed using the corresponding plasmids and incubating in medium containing 1 μg/ml of blasticidin.

#### Immunoblotting

Cells were lysed with SDS sample buffer (62.5 mM Tris–HCl [pH 6.8], 20% glycerol, 2% sodium dodecyl sulfate [SDS], 50 mM dithiothreitol) and electrophoresed in denaturing gels, and then transferred to polyvinylidene difluoride membranes. The membranes were blocked with 5% skimmed milk in PBS-T (PBS containing 0.05% Tween 20) for 30 min and reacted with the indicated antibodies for at least 1 h at room temperature or 4°C. The membranes were then reacted with secondary antibodies conjugated with peroxidase (Cytiva) and visualized using ECL with Fusion FX6.EDGE (Vilber Bio Imaging). The intensities of the indicated bands were evaluated using Image J software, and normalized to those of p65 or β-actin.^59^^,^^63^

#### Affinity precipitation

HEK293T cells were transfected with a plasmid expressing Strep-Flag-HHV-6B U28 or empty plasmid coupled with EGFP-p65. After 48 h, the cells were collected, and lysed with 0.5% NP-40 buffer (50 mM Tris-HCl [pH 8.0], 150 mM NaCl, 0.5% NP-40) containing a protease inhibitor cocktail (Nacalai Tesque). After centrifugation, the supernatants were reacted with MagStrep “type3” Strep-Tactin beads (IBA) with rotation for 2 h at 4°C. The precipitates were collected by brief centrifugation, washed extensively with 0.5% NP-40 buffer, and analyzed by immunoblotting.^54^^,^^59^

#### Luciferase assay

2 x 10^5^ HEK293T cells were co-transfected with pGL4.32 (NF-κB-luc; Promega) reporter plasmid and pRL-SV40 or pRL-TK together with an empty plasmid or expression plasmids encoding the indicated proteins.^31^ Luciferase activity was measured at 24 h post-transfection using a reporter assay system (Promega). The luciferase activity (Fluc/Rluc) was calculated as (firefly luciferase activity)/(*Renilla* luciferase activity). To analyze the effects of stimulation, the transfected cells were untreated or treated with 20 ng/ml TNF-α (PeproTech) from 24 h after transfection and incubated for another 4 h.

#### NanoBiT assay

4 x 10^5^ HEK293T cells were transfected with 500 ng of pSmBiT-vectors together with 500 ng of pLgBiT-vectors. After 24 h, Nano-Glo Live Cell Reagent (Promega) was added and luminescence was detected using a microplate reader.^56^

#### Immunofluorescence

For the transient experiments, HEK293T cells were transfected with the indicated plasmids using Lipofectamine 3000 for 48 h. To analyze infected cells, MT4 shLuc cells or MT4 shU28 cells were infected with HHV-6B and incubated for 3 days. These cells were then fixed with 4% paraformaldehyde for 10 min, permeabilized with 0.1% Triton-X100 for 10 min, and blocked with PBS containing 0.4% bovine serum albumin (BSA) for 30 min. These cells were stained with the indicated antibodies for 1 h at room temperature, followed by secondary antibodies conjugated to Alexa Fluor (Thermo Fisher Scientific) for another hour at room temperature. Finally, the cells were visually inspected using a LSM800 microscope (Zeiss).^59^^,^^63^^,^^64^ To determine viral infectivity, the infected cells were fixed with methanol/acetone and stained with mAb against gB. Nuclear DNA was stained with Hoechst 33342 and specific signals were detected using a fluorescence microscope (KEYENCE; BZ-X810).^55^

#### Live-cell imaging and FRAP assay

HEK293T cells were transfected with the indicated plasmids using Lipofectamine 3000 for 48 h. The cells were analyzed using an LSM800 microscope (Zeiss). To analyze fluorescence recovery after photobleaching (FRAP), HEK293T cells were transfected with the plasmid to express EGFP-NBR1 or EGFP-p65 and TagRFP-HHV-6B U28. The domains of EGFP-p65/TagRFP-HHV-6B U28 or EGFP-NBR1 were bleached for 2.0 s using a laser intensity of 30% at 488 nm, after which recovery was recorded for the indicated time.

#### Quantitative PCR (qPCR)

Total RNA was isolated from the cells with a NucleoSpin RNA kit (MachereyNagel) according to the manufacturer’s instructions, and cDNA was synthesized from the isolated RNA with SuperScript IV (Thermo Fisher Scientific). The amount of cDNA of specific genes was quantitated using the SYBR Select master mix (Thermo Fisher Scientific) according to the manufacturer’s instructions. The qPCR was performed on a QuantStudio 1 instrument (Thermo Fisher Scientific). The primer sequences were listed in Table S1. The amount of mRNA was normalized to the amount of β-actin mRNA. The relative abundance of each gene transcript was calculated using the comparative cycle threshold (2^−ΔΔCT^) method as described previously.^31^

#### Calculation of virus genome copy numbers

1 x 10^6^ MT4 cells were infected with HHV-6B. At 6 days after infection, DNA was extracted from the supernatant of the infected cells using the DNeasy Blood & Tissue Kit (QIAGEN), and the genome copy number per milliliter of infected cells was quantified by qPCR using SYBR Select master mix (Thermo Fisher Scientific). The primers used for this purpose were listed in Table S1.^31^^,^^55^

### Quantification and statistical analysis

RNA-seq library preparation and sequencing. 1x10^6^ MT4-shLuc or MT4-shU28 cells were treated with 20 μg/ml Dox for 24 h and then infected with HHV-6B HST for 72 h in the presence of the same concentration of Dox. Total RNA was isolated from the cells using Maxwell RSC simplyRNA cells kit (Promega) according to the manufacturer’s instructions. Under these conditions, about 20% of MT4 cells were found to be infected as determined by immunofluorescence. Sequencing libraries were generated using NEBNext Poly(A) mRNA Magnetic Isolation Module and NEBNext Ultra II Directional RNA Library Prep Kits (NEB) following the manufacturer’s recommendations. After cluster generation, the library preparations were sequenced on a NovaSeq 6000. Differentially-expressed genes (DEGs) were detected using DESeq2 (Version 1.24.0)^65^ with the threshold set at |log2FC (fold-change)| >1 and p.value <0.05. Heatmap was created by Heatmapper.^52^

#### Construction of a phylogenic tree

Amino acid sequences of viral RNR R1 proteins from all nine human herpesviruses were aligned using multiple-sequence comparisons, and phylogeny was constructed using a neighbor-joining tree without distance corrections and scaled for equal branch lengths (Genetycs ver. 2.2.5). Shaded boxes indicate herpesvirus subfamilies, which group closely to established phylogenetic trees.

#### Construction of alignment

Amino acid sequences of viral RNR R1 proteins from indicated herpesviruses were aligned using ClustalW by MEGA11.^51^ The RNR R1 homology region was determined by alignment of the nine human herpesvirus RNR R1s.

#### Statistical analysis

For two-group comparisons, the F-test was used to assess the equality of variances, and the two-tailed unpaired Student’s t-test or Welch’s t-test was used, depending on the equality of variances. Tukey’s test was used for multiple comparisons. A P value > 0.05 was considered not significant (n.s.). All statistical analysis was performed using GraphPad Prism 7 (GraphPad Software).

#### Data availability

RNA sequencing data have been registered in the DNA Data Bank of Japan and are publicly available as of the date of publication. Accession number is shown in the key resources table.
