# Supplementary material for: HHV-6B ribonucleotide reductase sequesters NF-κB subunit p65 to inhibit innate immune responses
Source: iScience. 2024 Dec 30;28(2):111710. doi: 10.1016/j.isci.2024.111710 (PMC11772975; doi:10.1016/j.isci.2024.111710)
Supplement: Document S1. Figures S1–S6 and Table S1 [file mmc1.pdf]

**iScience, Volume *28***

## Supplemental information

# HHV-6B ribonucleotide reductase sequesters NF-κB subunit p65 to inhibit innate immune responses

**Mansaku Hirai, Khoir Amaliin, Jing Rin Huang, Salma Aktar, Yasuko Mori, and Jun Arii**

## Supplemental figure legends

**Fig. Fig. S1 Roseolovirus U28 proteins inhibit NF-κB activation. Related to Figure 1.**

(A) Amino acid sequences of viral RNR R1 proteins from all nine human herpesviruses were aligned using multiple-sequence comparisons, and phylogeny was constructed using a neighbor-joining tree without distance corrections and scaled for equal branch lengths (Genetycs ver. 2.2.5). Shaded boxes indicate herpesvirus subfamilies, which group closely to established phylogenetic trees. The dotted box indicates genus Roseolovirus. HSV-1 UL39 (GenBank accession no. GU734771.1), HSV-2 UL39 (GenBank accession no. Z86099.2), varicella-zoster virus (VZV) ORF19 (GenBank accession no. CAA27910.1), HCMV UL45 (GenBank accession no. X04370.1), HHV-6A U28 (GenBank accession no. NC_001664.4), HHV-6B U28 (GenBank accession no. AB021506.1), HHV-7 U28 (GenBank accession no. AAC40751.1), EBV BORF2 (GenBank accession no. AJ507799.2) and Kaposi's sarcoma-associated herpesvirus (KSHV) ORF61 (GenBank accession no. AF148805.2). (B) HEK293T cells were transfected with the indicated expression plasmid or empty vector (EV). At 48 h post-transfection, the cells were analyzed by immunoblotting. β-actin was used for loading control. (C) The intensities of each protein staining in panel (E) in Fig. 1 normalized to β-actin are shown as means with standard deviations (n = 4; *, $P < 0.05$ [Welch's t-test]).

**Fig. S2. HHV-6B U28 induces p65 aggregates independent of phosphorylation of p65-S536. Related to Figure 2.** (A) Immunofluorescence microscopy images of HEK293T cells expressing EGFP-p65 or EGFP-p65 S536A and Flag-HHV-6B U28 or empty vector (EV) (upper panel). Those expressing EGFP-p65 or EGFP-p65 S536A and TagRFP-HHV-6B U28 or TagRFP were shown in the lower panel. The images are representative of the results of the experiment. Bars, 20 μm. (B) Percentage of Flag-HHV-6B U28-expressing cells (50-100 cells in each experiment) showing nuclear localization of EGFP-p65 and the cytoplasmic structures containing Flag-HHV-6B U28 and EGFP-p65. The data are means and standard deviations ($n = 4$; *, $P < 0.05$ **, $P < 0.01$; ***, $P < 0.001$ [Tukey's test]). (C) HEK293T cells were co-transfected with the NF-κB-luc reporter plasmid, pRL-TK, the indicated pEGFP-p65 and Flag-HHV-6B U28 expression plasmid or empty vector (EV). The luciferase activity was measured 24 h post-transfection. The data are shown as means and standard deviations ($n = 3$; **, $P < 0.01$ [Tukey's test]). (D) HEK293T cells were transfected with EGFP-NBR1 or EGFP-p65 and TagRFP-HHV-6B U28 and analyzed using FRAP assays. The images are representative of the results of the experiment. Bars, 2 μm. Images are representative of 3 independent experiments. (E) The mean fluorescence intensity of domains (n=6) was

quantified. The data are shown as means and standard deviations (n = 6; *, $P < 0.05$; **, $P < 0.01$ [Welch's t-test)]).

**Fig. S3. HHV-6B U28 has no effect on the subcellular localization of RIPK1 and NEMO. Related to Figure 2.** (A) HEK293T cells were co-transfected with HA-RIPK1 expression plasmid and plasmid to express Flag-HHV-6B U28 or tagRFP-HHV-6B U28 or empty vector (EV). After 48 h, the cells were observed under a confocal microscope after staining with the indicated antibodies. The images are representative of the results of the experiment. Bars, 20 μm. (B) Percentage of Flag-RNR-expressing cells (50-100 cells in each experiment) showing cytoplasmic structures containing EGFP-RIPK1 in experiments shown in panel (A). The data are shown as means and standard deviations (n = 3; ns, not significant. [Welch's t-test]). (C) HEK293T cells were co-transfected with the indicated plasmids and Flag-HHV-6B U28 or empty vector (EV). After 48 h, the cells were observed under a confocal microscope after staining with the indicated antibodies. The images are representative of the results of the experiment. Bars, 20 μm.

**Fig. S4. IκBα is not required for HHV-6B U28-mediated p65 inhibition. Related to Figure 3.** (A) HEK293T-shCT or -shIκBα cells were analyzed by immunoblotting. (B)

HEK293T cells were co-transfected with EGFP-p65 and TagRFP-HHV-6B U28 or TagRFP. After 48 h, the cells were observed under a confocal microscope after staining with anti-IκBα antibody. The images are representative of the results of the experiment. Bars, 20 μm.

**Fig. S5. U28 inhibits p65 activation during HHV-6B infection. Related to Figure 6.** (A) 1 x $10^5$ MT4 cells were either mock-infected or infected with HHV-6B HST. After 48 h, the cells were analyzed by immunoblotting with the indicated antibodies. (B) The intensities of pp65 in panel (A) normalized to p65. The data are shown as means and standard deviations (n = 3; *, P < 0.05 [Welch's t-test]). (C) 4 x $10^5$ MT4-shLuc or MT4-shU28 cells were infected with HHV-6B HST. After 96 h, the mRNA was collected from these cells. The expression of mRNA of *U28* was quantified by RT-qPCR. Relative amounts of these mRNAs were normalized to β-actin. The data are shown as means and standard deviations (n = 3; *, P < 0.05 [Welch's t-test]). (D) MT4-shLuc or MT4-shU28 cells were infected with HHV-6B HST. On day 6 post-infection, viral DNA was collected from these cells and quantified by qPCR. The data are shown as means and standard deviations (n = 4; ns, not significant. [Welch's t-test]). (E) The intensities of pp65 in each lane in panel (A) of Fig. 6, normalized to β-actin. The data are shown as means and

standard deviations (n = 3; **, P < 0.01 [Welch's t-test]).

**Fig. S6. Conservation of the N-terminal region of HHV-6B U28. Related to Figure 5.** (A) Schematics of RNR R1 proteins from the indicated herpesviruses. (B) Amino acid sequences of viral RNR R1 proteins from the indicated herpesviruses were aligned using ClustalW (MEGA11). The RNR R1 homology region was determined by alignment of the nine human herpesvirus RNR R1s. The residues shown in light blue are conserved in betaherpesviruses. The residues that are only conserved in roseoloviruses are shown in dark blue.

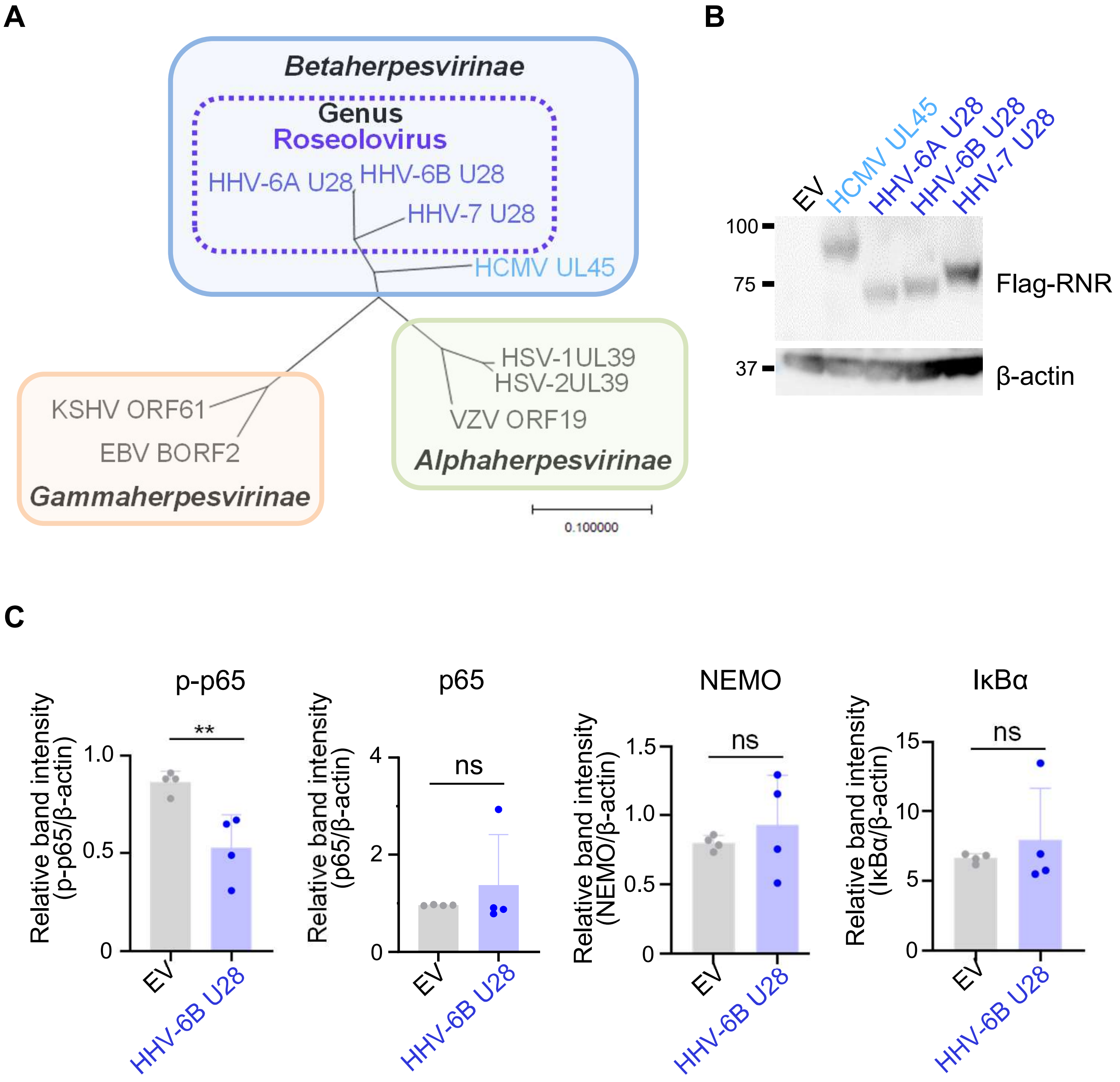


Fig. S1 Hirai et al.

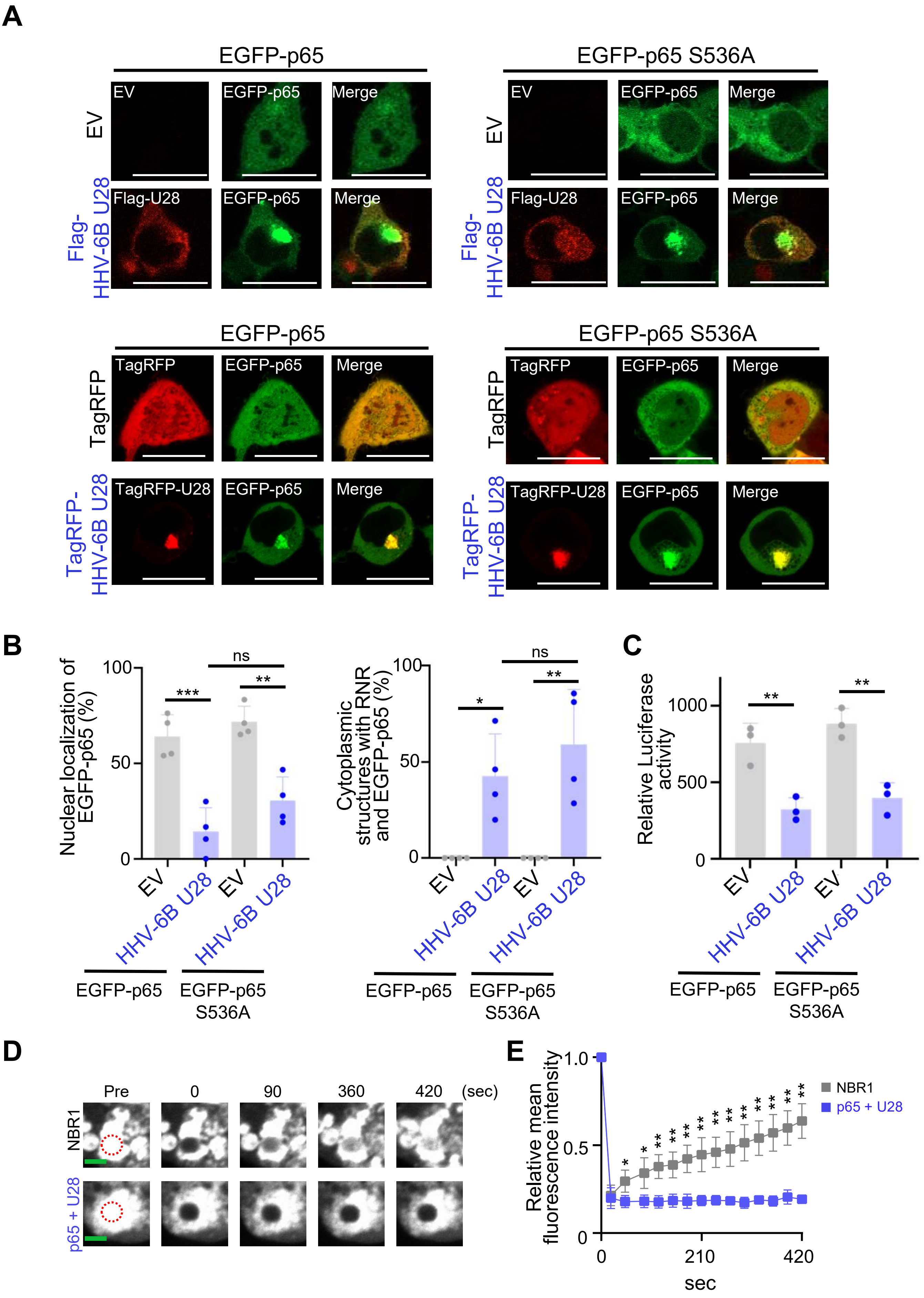


**Fig. S2 Hirai et al.**

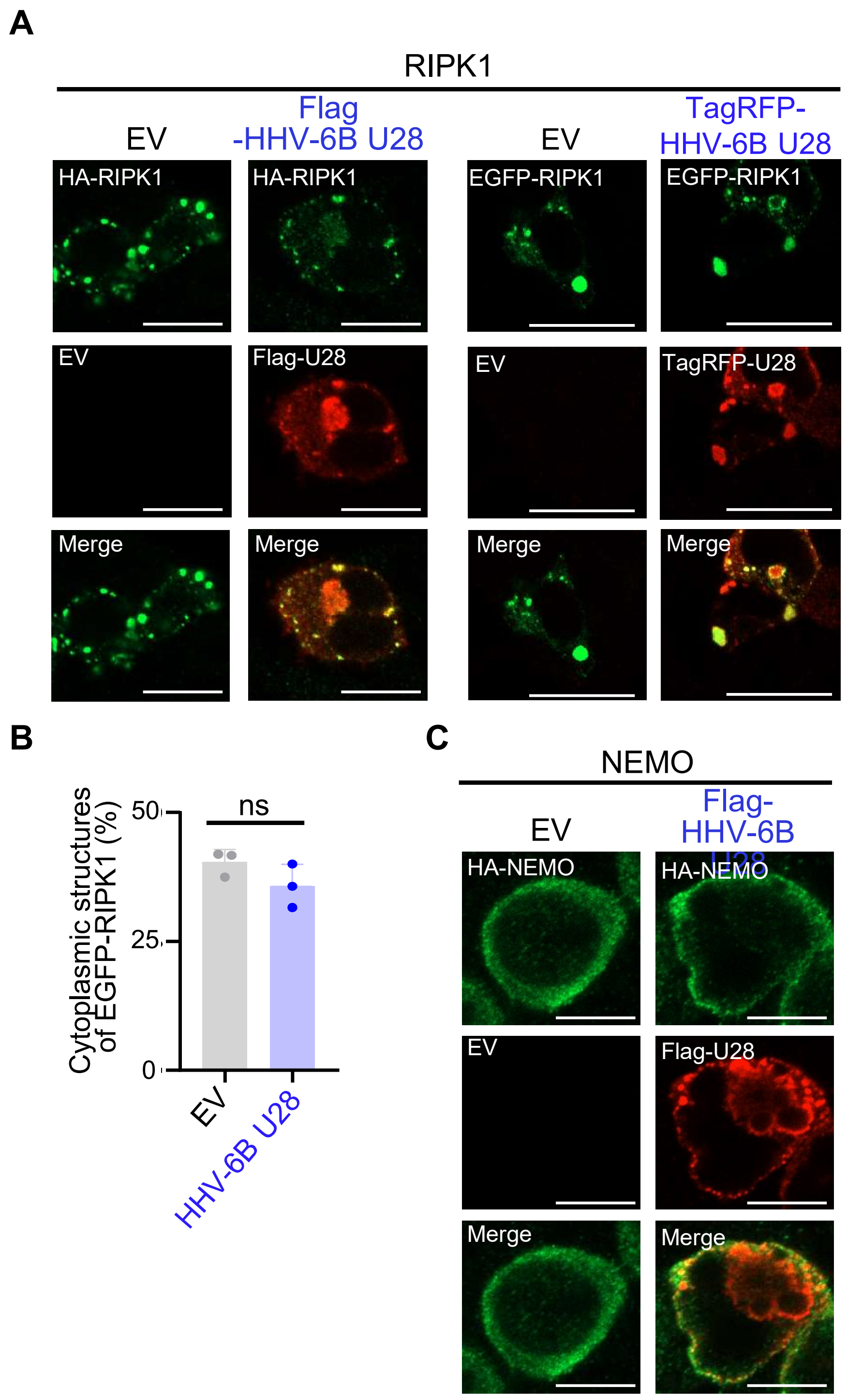


**Fig. S3 Hirai et al.**

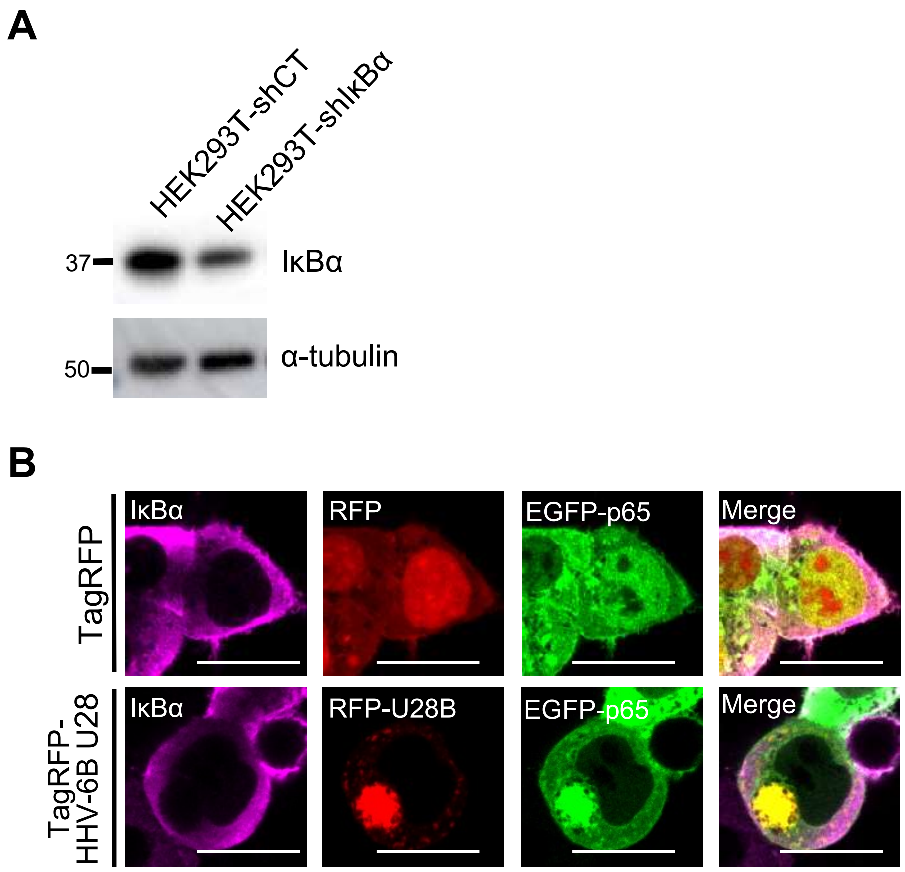


**Fig. S4 Hirai et al.**

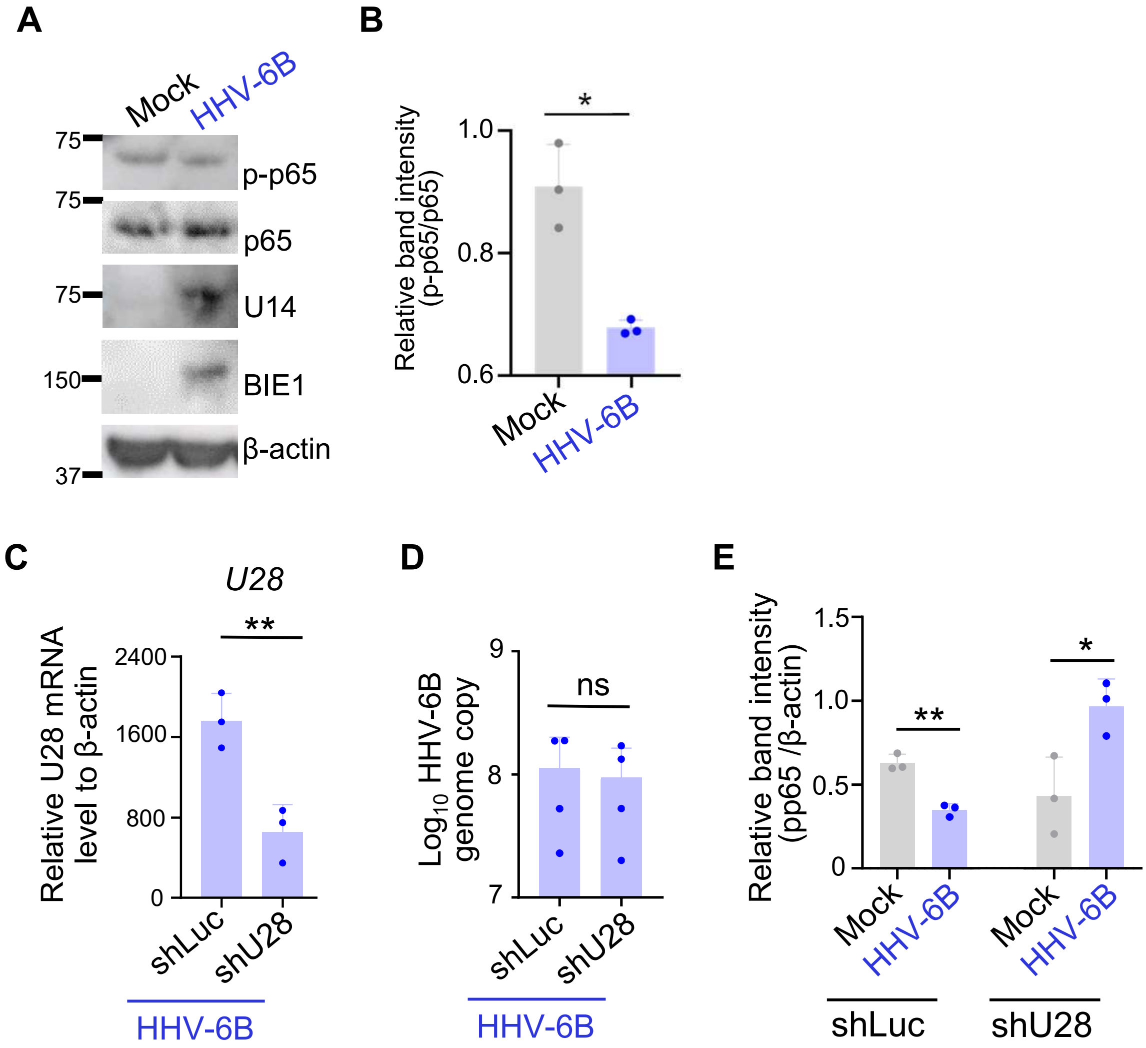


Fig. S5 Hirai et al.

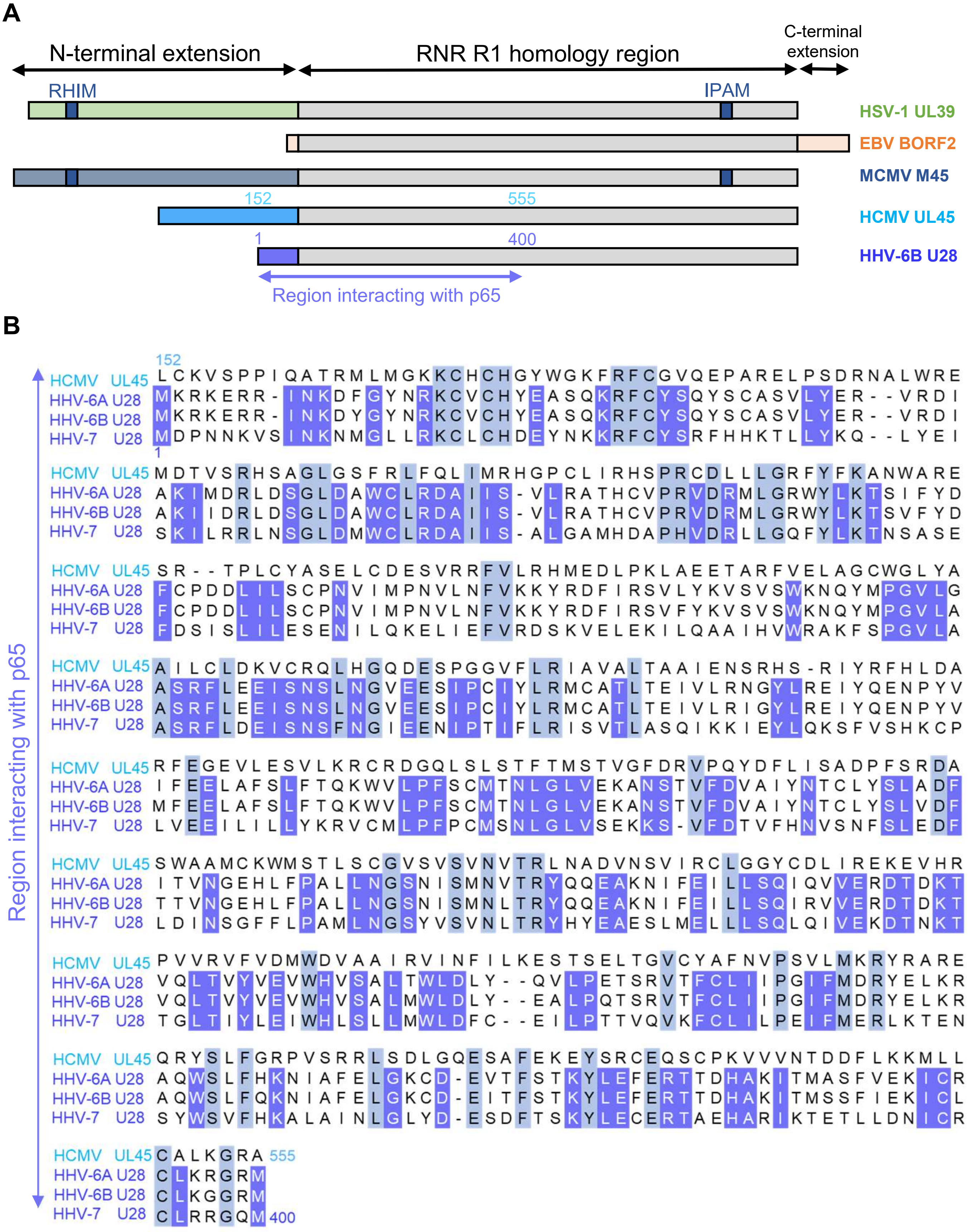


**Fig. S6 Hirai et al.**

**Table S1. Primer sequences. Related to STAR Methods.**

| Purpose | sequence |
|---|---|
| **RT-qPCR** | |
| IL-8 F | CTGGCCGTGGCTCTCTTG |
| IL-8 R | CCTTGGCAAAACTGCACCTT |
| β-actin F | GCACCCAGCACAATGAAG |
| β-actin R | CGATCCACACGGAGTACTTG |
| U28 F | ATGTGCCTTTGTATTGCGGG |
| U28 R | ATCGCTTACTTCGTCACCCA |
| **Calculation of virus genome copy numbers** | |
| HHV-6 genome F | CAAAGCCAAATTATCCAGAGCG |
| HHV-6 genome R | CAAAGCCAAATTATCCAGAGCG |
| **Cloning of pInducer10 vector** | |
| shLuc | TGCTGTTGACAGTGAGCGCAGGAATTATAATGCTTATCTATAGTGAAGCCACAGATGTATAGATAAGCATTATAATTCCTATGCCTACTGCCTCGGA |
| shU28 | TGCTGTTGACAGTGAGCGCGTATAAACACCTTGATTTATCTAGTGAAGCCACAGATGTAGATAAATCAAGGTGTTTATACATGCCTACTGCCTCGGA |
| pInd10-F | CAGAAGGCTCGAGAAGGTATATTGCTGTTGACAGTGAGCG |
| pInd10-R | CTAAAGTAGCCCCTTGAATTCCGAGGCAGTAGGCA |
| **Cloning of pLKO vector** | |
| IκBα-F | CCGGAGAGTCAGAGTTCACGGAGTTCTCGAGAACTCCGTGAACTCTGACTCTTTTTTG |
| IκBα-R | AATTCAAAAAAGAGTCAGAGTTCACGGAGTTCTCGAGAACTCCGTGAACTCTGACTCT |
